# The Hospital Library and Charities Bureau

**Published:** 1901-10-05

**Authors:** 


### The Institutional Workshop.

### THE HOSPITAL LIBRARY AND CHARITIES BUREAU.

There has just been opened in the Hospital Building, 28 & 29 Southampton Street, an institution which, we hope, may prove to be of considerable value to the ever-increasing number of persons who are either connected with, or interested in, hospitals and general charities. The Hospital Library and Charities Bureau, to which allusion has already been made in our columns, has been established by Sir Henry Burdett, assisted by generous friends. It is intended to be a centre of reference for this class just in the same manner as the Colonial Institute is for the people who are specially interested in all the details of movements in the colonies. And first as to the material provided for those who become members of the library. To accumulate it has been the labour of many years, and of much patience. It has involved great research, and, of course, considerable expense. Under the direction of Sir Henry Burdett, the reports, rules, and regulations of the hospitals and charitable institutions, not only of Great and of Greater Britain, but of the world, have been collected, bound, and catalogued, and to these have been added plans of the more important hospitals and new buildings. The result is that there now exists a complete financial and constructional history of many hospitals and societies of all kinds. The library already contains all the standard works on hospital administration, construction, finance, statistics, and nursing, but it is proposed to enrich it periodically with such books as may be published on any of these subjects, and thus to keep it absolutely up to date.

### The Bureau.

The Bureau is a unique feature. Members will be able, on application to the librarian, to obtain expert opinion on any questions they require to be answered, either gratuitously (by the kind permission of the Editors, through the pages of The Hospital), or on payment of a small fee, by letter. That the mass of available and accurate information will be found widely useful we cannot doubt.

### The Reference Library.

There is no one who has not something to learn, and no institution whose system of management is perfect. Even medical men of ripe experience may be glad to add to their knowledge of administration by consulting the library. Secretaries of Hospitals, and managers of charitable enterprises who, if they are anxious to keep abreast with the times, lose no opportunity of becoming acquainted with what is going on outside their own little circle, will doubtless avail themselves of the easiest possible method of ascertaining the principles upon which kindred institutions to their own are conducted, and turn to practical account the successful points of other systems.

### The Collection of Plans.

For architects, to whom the problem of construction, or reconstruction, is constantly presenting itself for solution, the exhaustive plans in the library constitute quite a mine of wealth, and will repay the most careful and continued study. There are hopelessly inconvenient and wretchedly-planned hospitals in existence which would never have been erected if the architects had only possessed the facilities of enlightenment which are now offered to them. The library is established for the use of both sexes, and matrons of hospitals will profitably occupy an occasional hour in mastering the rules and regulations which obtain in the leading training schools beyond the seas. The cottage hospital, which is rapidly becoming indispensable in the smallest of towns and the largest of villages, claims the special attention of the clergy, and many a country vicar who is the chairman of a committee charged with the task of founding one, will get rid of his perplexities about the cost of building and equipment by a few inquiries at the bureau.

### For the General Public.

The library must also appeal with irresistible force to the kindly philanthropist meditating the bestowal of a proportion of his wealth on some deserving project, but fearful lest he should throw away his money on one which is not worthy of help. If he is a member of the Hospital Library he will be in a position to readily arrive at a satisfactory conclusion. We have mentioned the Colonial Institute, and our colonial cousins who are interested in hospitals and charities at home may rejoice when they are visiting the Mother Country, to profit by the chance of belonging to a library which places within their grasp information that will enable them on their return to suggest, or effect, improvements in their own institutions.

The following arrangements have been made and will give an idea of the scope of the scheme, and information regarding the fees for membership :—

1. The Hospital Library and Bureau at 28 and 29 Southampton Street, Strand, W.C., is open to members from 10 to 5 daily, except on Saturdays, when it is closed at 1 o'clock.

2. The library is at the disposal of all engaged or interested in hospital nursing or institutional work, both ladies and gentlemen.

3. The library is a reference library, and must only be used for this purpose.

4. The fee for membership is : gentlemen, 10s. 6d. ; ladies, 5s. per annum. Non-members are allowed to use the library on payment of 2s. 6d. for each occasion. Members of the Hospitals Association are entitled to the free use of the library and all privileges in connection therewith without any payment. (The subscription for membership of the Hospitals Association is £1 1s. per annum for gentlemen, 10s. 6d. per annum for ladies.)

5. Members have the privilege of submitting in writing any questions upon which they may require information, and these questions, so far as practicable, will be answered in the pages of The Hospital. Non-members may submit questions personally or by letter upon payment of a fee of 2s. 6d. for each completed inquiry.

6. A book is kept in the library in which members can enter the names of any works which they may think it desirable to add to the library. This list will be periodically gone through, and such books as may be considered of sufficient general interest will be procured from time to time.

